# Systemic, Mucosal, and Memory Immune Responses following Cholera

**DOI:** 10.3390/tropicalmed6040192

**Published:** 2021-10-27

**Authors:** Edward T. Ryan, Daniel T. Leung, Owen Jensen, Ana A. Weil, Taufiqur Rahman Bhuiyan, Ashraful Islam Khan, Fahima Chowdhury, Regina C. LaRocque, Jason B. Harris, Stephen B. Calderwood, Firdausi Qadri, Richelle C. Charles

**Affiliations:** 1 Division of Infectious Diseases, Massachusetts General Hospital, Boston, MA 02114, USA; etryan@mgh.harvard.edu (E.T.R.); rclarocque@mgh.harvard.edu (R.C.L.); jbharris@mgh.harvard.edu (J.B.H.); scalderwood@mgh.harvard.edu (S.B.C.); 2 Department of Medicine, Harvard Medical School, Boston, MA 02115, USA; 3 Department of Immunology and Infectious Diseases, Harvard T.H. Chan School of Public Health, Boston, MA 02115, USA; 4 Division of Infectious Diseases, Department of Internal Medicine, University of Utah School of Medicine, Salt Lake City, UT 84132, USA; daniel.leung@utah.edu (D.T.L.); owen.jensen@path.utah.edu (O.J.); 5 Division of Allergy and Infectious Diseases, University of Washington, Seattle, WA 98109, USA; anaweil@uw.edu; 6 International Centre for Diarrhoeal Disease Research, Bangladesh (icddr,b), Dhaka 1212, Bangladesh; taufiqur@icddrb.org (T.R.B.); ashrafk@icddrb.org (A.I.K.); fchowdhury@icddrb.org (F.C.); fqadri@icddrb.org (F.Q.); 7 Department of Pediatrics, MassGeneral Hospital for Children, Boston, MA 02114, USA; 8 Mucosal Immunology and Biology Research Center, Division of Pediatric Gastroenterology and Nutrition, Massachusetts General Hospital, Boston, MA 02115, USA; 9 Division of Pediatric Global Health, Massachusetts General Hospital, Boston, MA 02115, USA; 10 Department of Microbiology, Harvard Medical School, Boston, MA 02115, USA

**Keywords:** cholera, *Vibrio cholerae*, immunity, innate, adaptive, antibody, cellular, mucosal, systemic, memory, vaccine

## Abstract

*Vibrio cholerae* O1, the major causative agent of cholera, remains a significant public health threat. Although there are available vaccines for cholera, the protection provided by killed whole-cell cholera vaccines in young children is poor. An obstacle to the development of improved cholera vaccines is the need for a better understanding of the primary mechanisms of cholera immunity and identification of improved correlates of protection. Considerable progress has been made over the last decade in understanding the adaptive and innate immune responses to cholera disease as well as *V. cholerae* infection. This review will assess what is currently known about the systemic, mucosal, memory, and innate immune responses to clinical cholera, as well as recent advances in our understanding of the mechanisms and correlates of protection against *V. cholerae* O1 infection.

## 1. Introduction

Cholera is a severe dehydrating disease of humans caused by *Vibrio cholerae* serogroup O1 and O139. Over one billion people remain at risk for cholera in 51 endemic countries, and there are an estimated three million cases and 95,000 deaths from the disease each year [1]. The current global cholera pandemic began in 1961 with El *Tor V.cholerae* O1 and shows no signs of abating, as evidenced by recent large outbreaks in Haiti, Yemen, and South Sudan and annual epidemics in countries in Asia and Africa. This reality has led to enhanced commitments to cholera control strategies [2]. Such strategies now include vaccination against cholera, as well as improved water and sanitation efforts [2]. Currently available oral killed-cholera vaccines (kOCVs) have been a transformative addition to these control efforts; however, these vaccines may provide limited durable protection, especially in immunologically naïve individuals, including children under five years of age who bear a large proportion of the global cholera burden [3]. In comparison, survivors of clinical cholera, including young children, have high-level protective immunity that persists for years [4]. An improved understanding of immune responses associated with protection against cholera could lead to next-generation vaccines or prevention strategies. This review will assess what is currently known about the systemic, mucosal, memory, and innate immune responses to clinical cholera, as well as recent advances in our understanding of the mechanisms and correlates of protection against *V. cholerae* O1 infection

## 2. *V. cholerae*-Antigen Repertoire

### V. cholerae O-Specific Polysaccharide (Lipopolysaccharide)

Protection against cholera is serogroup-specific, and serogroup specificity is dictated by the O-specific polysaccharide (OSP) component of *V. cholerae* lipopolysaccharide (LPS). Because of this, there is no cross-protection between infection with *V. cholerae* O1 and O139, even though these organisms can both cause epidemic cholera and are essentially genetically identical except for differences in the *rfb* genes encoding the OSP of these two serogroups [5,6,7]. Antibodies that bind externally to *V. cholerae* are binding to surface displayed antigens, either outer membrane proteins or LPS. Previous work has shown that the vibriocidal response is mediated by antibodies that bind to LPS, and specifically OSP [8,9]. Following clinical cholera, over a third of all induced antibodies target *V. cholerae* OSP [10,11]. These data would suggest that anti-LPS and specifically OSP-specific immune antibody responses are the actual mediators of protection against cholera.

Before OSP became available as a reagent for use in immunologic assays, a body of evidence showed that LPS responses (plasma, mucosal and memory) occur following cholera and vaccination in both children and adults, and that these responses correlated with protection against cholera, including in young children [12,13,14,15]. These findings were confirmed with OSP, once it became available for study [9,16,17,18,19]. Anti-OSP/LPS IgG, IgA and IgM responses following immunization of children in Bangladesh with killed oral cholera vaccines are significantly lower than those induced following clinical disease in age-matched patients, including the absence of anti-LPS memory responses in vaccinees despite induction of vibriocidal responses [15,16,18]. Specifically, infants and young children receiving kOCVs did not mount IgG, IgA, or IgM antibody responses to *V. cholerae* OSP or LPS, whereas older children showed significant responses.

In comparison to the vaccinees, young children with wild-type *V. cholerae* O1 infection showed significant antibody responses against OSP/LPS. OSP responses correlated with age in vaccinees, but not in cholera patients, reflecting the ability of even young children with wild-type cholera to develop OSP responses. These differences might contribute to the lower efficacy of protection rendered by kOCV than by wild-type disease in young children and suggest that efforts to improve OSP-specific responses might be critical for achieving optimal cholera vaccine efficacy in this younger age group [15,16]. In addition, avidity of anti-LPS IgG and IgA antibodies following wild-type disease is high and prolonged, despite a decrease in vibriocidal titers by day 180; and anti-LPS avidity correlates with induction of memory B-cell responses [20]. Anti-LPS avidity falls rapidly to baseline by day 30 following oral vaccination, suggesting a possible explanation for lower and shorter-term immunity afforded by kOCVs [20]. These data suggest that LPS/OSP specific responses may be better markers of long-term protection against cholera in endemic zones than other immune responses.

Much effort is now being made to assess how OSP-specific antibodies might protect against cholera, with a growing body of evidence suggesting that protection against infection may involve the ability of OSP-specific antibodies to impede the motility of *V. cholerae* organisms in the human intestine. This effect requires at least two-point binding of OSP-specific antibodies [21,22,23,24,25,26].

## 3. Protein Antigens

In addition to OSP, well-characterized *V. cholerae* antigens include the following proteins: cholera toxin B subunit (CTB), the toxin co-regulated pilus (TCP) subunit A (TcpA), and *V. cholerae* cytolysin (VCC), also referred to as hemolysin A (HlyA). Cholera toxin is a major virulence factor for all toxigenic strains of *V. cholerae* and consists of five B (CTB) subunits associated non-covalently with a single, enzymatically active A subunit [27]. TcpA is a major structural component of TCP, a colonizing factor essential for virulence in humans [28]. VCC generates membrane pores in eukaryotic cells and has been shown to induce fluid accumulation in ligated rabbit ileal loops and induce chloride secretion in intact human intestinal mucosa [29,30]. CTB, TcpA, and VCC have been shown to induce systemic, mucosal, and memory B-cell responses after *V. cholerae* infection [17,31,32,33].

Additional antigenic targets of the immune response to *V. cholerae* have recently been identified. The antigenic repertoire recognized by the cholera-induced plasmablast population was assessed through generation of a panel of monoclonal antibodies (mAbs) isolated following single-cell expression of day 7 plasmablasts from *V. cholerae*-infected patients [11]. Plasmablasts are activated antibody-secreting cells that are transiently found in the circulation after either infection or vaccination. Both cholera and cholera vaccines induce a potent mucosal homing plasmablast response that peaks seven days after infection and is strongly predictive of the presence of specific duodenal plasma cells for up to six months after cholera [10,34], suggesting that a proportion of these cells ultimately take up residence in the intestine as plasma cells. Of the 138 mAbs that were generated from a total of seven participants, 24 were OSP-specific, 37 were CtxB-specific, 12 were CtxA-specific, and none were TcpA-specific [11]. Using a *V. cholerae-*antigen array containing 95% of the *V. cholerae* proteome, nine additional antigenic targets were identified, most notably *V. cholerae* sialidase/mucinase (NanH), which was the target of 6 mAbs (or 5% of all circulating plasmablasts). Flagellin protein A and ToxR-regulated mucinase tagA were also identified.

This work was further supported by an immunoscreen using the *V. cholerae* antigen array above with plasma and antibodies recovered from culture supernatants of activated plasmablasts [35]. Fifty-nine antigens were demonstrated to have higher immunoreactivity at the early convalescent stage of infection compared to the acute stage or healthy controls [17]. These included the known antigens OSP, CTB, TcpA, VCC, as well as several novel antigens, including NanH, cholera toxin A subunit (CtxA), an outer membrane protein (OmpV), a protein phosphotransferase (PtsP), and flagellar proteins (FlaC, FlaD).

Systemic, mucosal, and memory B-cells responses to NanH occur after clinical cholera, with NanH found to be the third most common antigenic target of a mucosal homing plasmablast response [11,17,36]. NanH is a virulence factor that catalyzes the cleavage of terminal sialic acid residues from gangliosides on the membrane of intestinal epithelial cells to generate monogangliosides (GM1), the binding site for CT [37]. NanH-neutralizing antibodies have been shown to block the toxin-potentiating effect of NanH in vitro [11]. These findings together suggest a possible functional role for NanH in protective immunity to cholera and antibody responses to NanH have been found to correlate with protection [36].

## 4. Correlates of Protection

### 4.1. Vibriocidal Response-Pros/cons

The vibriocidal response has historically been used to assess protection against cholera, but antibody-based bacterial killing of *V. cholerae* in the intestinal lumen is unlikely. There is no evidence that enhanced opsonophagocytosis or antibody-dependent cytotoxic activity in the intestinal lumen plays a role in mediating protection against cholera. Although cell-free antibody-based killing via complement lysis might be considered possible in the intestinal lumen, viability studies in animals have shown that bactericidal activity is not required for protection from disease [22,25,26]. In addition, although C3 and earlier components of the complement cascade have been detected in the intestinal lumen/epithelium, the terminal components of the complement cascade have not been detected in the lumen in the absence of epithelial breakdown and intestinal inflammation [38,39,40]. The primary antibody secreted at mucosal surfaces is IgA; however, IgA lacks the Fc regions of IgG and IgM that bind C1q, and consequently does not activate complement via the classical complement pathway; moreover, IgA complexed to antigen actually inhibits complement activation by IgM and IgG [41,42,43]. These data suggest that IgA plays a minimal, if any, role in complement activation in the intestine. The vibriocidal antibody assay, however, assesses complement activation [12,44,45].

While the vibriocidal response is associated with protection against cholera [46] and is an important biomarker of recent infection [47], there is no absolute value above which protection is assured [44]; and cholera vaccines that have been equivalent to wild type infection in inducing vibriocidal antibody responses have failed in clinical field trials in humans [48]. Similarly, although vibriocidal antibody titers increase sharply within 10 days of symptomatic cholera, they then fall rapidly within 30 days of infection, returning to baseline within 6–12 months, despite the fact that an episode of symptomatic cholera induces long-term immunity against cholera that exists for at least three to 10 years [4,49,50]. In addition, individuals can be protected against cholera with no increase of vibriocidal antibody responses following challenge and exposure, suggesting that other immune responses mediate protection against cholera [50,51,52,53]. Importantly, OSP responses differ substantially in naïve North Americans and low-to-middle-income country residents infected with *V. cholerae*, despite induction of comparable vibriocidal responses [54]. These data strongly suggest that the vibriocidal antibody is at best an imperfect non-mechanistic correlate of protection against cholera.

The majority of the vibriocidal response consists of IgM antibodies that specifically target OSP, giving the vibriocidal its serogroup specificity [9]. Interestingly, IgM antibodies, just like IgA antibodies, are actively transported across intact intestinal epithelium into the intestinal lumen, so it is quite possible that anti-vibrio OSP IgM antibodies may play a role in mediating at least short-term protection against cholera (for instance, following vaccination). Still, there are no data that this protection is mediated by complement-dependent membrane attack complex (MAC)-based lysis at a mechanistic level. If the vibriocidal antibody response is only a surrogate/correlate of protection against cholera, is there a related mechanistic antibody response that might actually mediate protection, especially long-term protection not afforded by IgM? Mucosal IgA and IgM antibodies may protect against pathogens at mucosal surfaces by inhibiting bacterial-epithelial cell interactions (“immune exclusion”), and/or via bacterial “clumping” either via agglutination at high bacterial concentrations (≥10^8^ non-motile bacteria per gram), or “enchaining growth” (antibody-mediated cross-linking preventing bacterial separation after bacterial division) at lower bacterial concentrations, with facilitated mucosal clearance of clumped bacteria [55]. Recent data also suggest that inhibition of motility of *V. cholerae* in the intestinal lumen may contribute mechanistically to protection against cholera [25,26].

### 4.2. Memory B-Cells

Individuals with cholera are protected for a period well beyond when the serum vibriocidal antibody, circulating antigen-specific plasmablasts, and serum antibody to specific cholera antigens have disappeared or waned in the circulation, suggesting that longer-term protection may depend on anamnestic responses mediated by immunologic memory following primary infection. During primary infection, naïve B-cells traffic through secondary lymphoid tissues, where the B-cell receptor may recognize an antigen presented on an antigen-presenting cell, priming that B-cell to internalize, process, and present that antigen on MHC class II molecules. Primed B-cells interact with primed T follicular helper cells, a subset of CD4-positive T-cells that have been similarly primed from a naïve T-cell by interaction with the same specific antigen presented on an antigen-presenting cell. The interaction of the primed B and T follicular helper cells activate the B-cell to undergo further proliferation, somatic hypermutation, and isotype switching, and subsequently to differentiate into memory B-cells and long-lived plasma cells specific for that antigen, which mediate immunologic memory [56].

Both adults and children with cholera develop memory B-cells of the IgG and IgA isotypes specific to protein antigens, such as CTB and TcpA, which persist in the circulation out to at least one year following infection. Sialidase-specific IgA memory B-cells have also been demonstrated after cholera [36]. Similarly, memory B-cells also develop that recognize both LPS and the OSP of *V*. *cholerae* O1, which persist in the circulation out to at least 90-180 days following infection [15,32,33,47,57]. The mechanisms by which isotype-switched memory B-cells develop to the T-cell-independent antigens LPS and OSP are currently unknown.

Individuals receiving kOCVs develop memory B-cells to CTB and OSP, but at substantially lower levels than seen with natural infection and are shorter-lived [15,57,58]. These differences might contribute to the lower efficacy of protection rendered by these vaccines. The live-attenuated cholera vaccine, CVD 103-HgR (approved in the United States as a traveler’s vaccine), has been shown in clinical trials in developed countries to induce vibriocidal responses that persist beyond two years in older children [59] and beyond one year in adults [60]. In addition, induction of memory B-cells to LPS and CTB has also been demonstrated in a US population [60,61]. However, oral vaccines (including CVD 103-HgR) induce lower immune responses and efficacy in resource-poor countries compared to developed countries [62]. More studies of live attenuated vaccines are needed in endemic countries as several host factors are suspected to impact responses in different geographic populations including nutritional status, intestinal epithelial barrier integrity, enteric enteropathy, concurrent infection, and diet [62,63,64,65,66]

Using a household contact study approach, OSP responses were found to correlate with protection in cholera endemic populations, including in young children [57]; and OSP and LPS responses were found to correlate with protection against cholera in an experimental infection model in humans [67,68]. However, no protection was mediated by the presence of circulating memory B-cells recognizing CTB [14,57]. There is currently inadequate data on the presence and persistence of antigen-specific memory B-cells or long-lived plasma cells in gastrointestinal mucosa and whether these might mediate more direct local protection from infection or disease.

## 5. Innate Immune Responses to Cholera

Microscopic characterization of intestinal mucosal tissue obtained by endoscopic biopsy shows that *V. cholerae* disrupts components of the epithelium and is associated with an influx of inflammatory cells, including neutrophils, lymphocytes and macrophages, into the lamina propria [69,70]. At a molecular level, early changes include the increased expression of a wide array of antibacterial effector proteins such as lactoferrin (LTF), lipocalin2 (LCN2) and Bactericidal/Permeability-increasing-fold-containing family B member 1 (BPIFB1; also known as LPLUNC1), as well as oxidases including nitric oxide synthase (NOS2) and dual oxidase 2 (DUOX2) [71,72,73]. *V. cholerae* infection also results in the upregulation of key cytokine signaling hubs. Several of these activated signaling pathways, such as the NLR family pyrin domain containing 3 (NLRP3) inflammasome and interferon regulatory factor 7 (IRF7)/type I interferon, are not typical of the innate immune response to pyogenic bacterial infections and are instead more typical of the response seen with viral infection [73].

The functional significance of the innate immune responses to *V. cholerae* infection is not fully understood. While the innate immune system does not entirely prevent infection—since most immunologically naïve individuals who ingest enough organisms will become infected—it nonetheless plays an essential role in directing the adaptive immune response. For example, the innate immune response to live pathogenic *V. cholerae* results in the expression of key cytokines such as IL-23 that promote B- and T-cell differentiation [74].

The innate immune system may also impact disease severity. For example, human biopsy-derived enteroids (an in vitro model for the intestinal epithelium) engineered to express the human blood group O-antigen are more susceptible to the effects of cholera toxin than enteroids expressing the A-type antigen [75]. This provides a molecular basis for the link between the blood group O phenotype and increased disease severity, and also may explain why the lowest prevalence of the O blood group phenotype in the world is observed the Bengal region of South Asia, where cholera is historically endemic [76]. Similarly, a genome-wide association study found that variations in both the type I interferon and NLRP3 inflammasome signaling pathways have been under strong selective pressure in Bangladesh, suggesting that these cholera-linked innate immune responses have played an important role in human survival historically in this region [77].

## 6. Interaction between Microbiota and Cholera Immunity

The composition of the gut microbiota may also impact susceptibility to *V. cholerae* infection, the clinical severity of disease, and subsequent immune responses. For the identification of microbial markers associated with susceptibility to infection, a recent study characterized the microbiota of close contacts of cholera patients in Bangladesh [78] by measuring the gut microbiota at the time of a shared exposure to the household case or common water source. The study assessed the microbial composition before and after exposure and found that the gut microbiota predicted susceptibility to infection at least as well as the clinical risk factors known to contribute to susceptibility, such as age, baseline vibriocidal titer, and blood group O-status.

Several taxonomic groups, such as *Enterobacteriaceae* and *Streptococcus,* were correlated with the stool of individuals who developed infection. At the same time, the genera *Prevotella*, *Bacteroides,* and *Lactobacillus* were more dominant among contacts who did not develop infection during the follow-up period. Using the same cohort of household contacts, a metagenomic study was conducted that identified specific gene groups that correlated with the development of infection [79]. For example, iron metabolism and regulation genes were more common in the gut microbiota of persons who did not develop infection after exposure. Like most bacteria, *V. cholerae* has several mechanisms of scavenging iron in the gut, and defects in iron metabolism have been shown to be critical to *V. cholerae* virulence in animal studies [80,81,82]. Bile acids are also metabolized by the gut microbiota and may impact susceptibility to *V. cholerae* infection. The bacterium *Blautia obeum* has been found to impact the ability of *V. cholerae* to colonize an animal host by degrading taurocholate, a conjugated bile acid [83]. *V. cholerae* senses taurocholate in the small intestinal environment and this triggers virulence factor expression; however, in the presence of *B. obeum*, this effect is reduced [83,84]. These studies demonstrate that commensal microbes in the human gut microbiota may potentially impact susceptibility to *V. cholerae* infection, through signaling molecules or other mechanisms. They may also impact responses to oral cholera vaccination. This remains an area of active study.

## 7. Mucosal-Associated Invariant T (MAIT) Cells

Mucosal-associated invariant T (MAIT) cells are innate-like T-cells defined by the expression of an invariant T-cell receptor (TCR) alpha chain, Vα7.2 (TRAV1-2) in humans, with a limited diversity of TCR beta chains [85,86,87]. The MAIT TCR recognizes microbial-derived metabolites of the riboflavin biosynthetic pathway presented on the MHC Class 1-related protein, MR1 [88]. MAIT cells make up approximately 1-10% of T-cells in healthy human blood, and are enriched in the liver, skin, lymph nodes and intestinal and respiratory mucosa [86,89,90,91,92,93]. MAIT cells are potent producers of pro-inflammatory cytokines and cytotoxic molecules and have been shown to provide protection against mucosal bacterial pathogens in murine challenge models [94,95,96,97]. Given their potential to participate in both innate and adaptive immunity, MAIT cells have been postulated to play key roles in mucosal infections. Observational studies have shown that MAIT cell frequency is reduced in the blood of humans with mucosal infections [98,99,100,101]. This finding, along with an increase in activation and gut homing markers [98,102,103] suggests that MAIT cells may traffic to the mucosa during infection. Despite their enrichment in the human gastrointestinal (GI) tract, knowledge about MAIT cells in the context of GI bacterial infections remains limited.

*V. cholerae* is capable of de novo riboflavin biosynthesis using the riboflavin biosynthetic pathway [104]), the intermediates of which can activate MAIT cells [105]. Flow cytometric analysis of MAIT cells in Bangladeshi adult and pediatric patients presenting with severe culture-confirmed cholera showed that at seven days post-cholera onset, peripheral blood MAIT cells had increased expression of activation markers, and MAIT frequency was significantly decreased in pediatric but not adult patients, suggesting potential trafficking to the mucosa [106]. In support, analysis of duodenal biopsies and peripheral blood MAIT cells from another cohort of adult Bangladeshi cholera patients revealed increases in duodenal lamina propria MAIT cell frequency and expression of gut homing markers in peripheral blood MAIT cells [107]. Changes in frequency of peripheral blood MAIT cells were also found to correlate with LPS IgA and IgG responses, suggesting that MAIT cells may be associated with class switching for T-cell-independent antigens [106]. Furthermore, in a murine model of *V. cholerae* intranasal vaccination in T-cell deficient mice, adoptive transfer of MAIT cells rescued *V. cholerae*-specific IgA responses and promoted B-cell differentiation [108]. Although these data highlight that MAIT cells, especially those in the duodenal mucosa, are activated in response to *V. cholerae* infection, further investigation into their function and phenotype is necessary to understand the role that MAIT cells play in cholera immunity.

## 8. Conclusions

Our understanding of cholera immunity has greatly increased over the last two decades. However, gaps remain in our understanding of the primary mechanisms of protective immunity to cholera, specifically whether antigen-specific memory B-cells or long-lived plasma cells are present and persist in gastrointestinal mucosa and the various physiologic mechanisms of an effective antibody-mediated response against cholera. In addition, further assessment of how the gut microbiome and innate immune response modulate the adaptive immune response to infection and vaccination will be important for a full understanding cholera immunity. These insights into the variation in immune responses between natural infection and vaccination will lead to improved vaccination strategies.
